# Case Report: Successful treatment of steroid- and ruxolitinib-refractory gastrointestinal acute graft-versus-Host disease with anti-thymocyte globulin

**DOI:** 10.3389/fimmu.2025.1610071

**Published:** 2025-07-25

**Authors:** Lulu Zhang, Fan Wu, Huiping Wang, Zhimin Zhai, Lili Tao

**Affiliations:** Department of Hematology, The Second Affiliated Hospital of Anhui Medical University, Hefei, China

**Keywords:** allogeneic hematopoietic stem cell transplant (HSCT), acute GVHD, gastrointestinal acute GVHD, anti-thymocyte globulin, graft-versus-host

## Abstract

Acute graft-versus-host disease (aGVHD), particularly with gastrointestinal (GI) involvement, remains a life-threatening complication after allogeneic hematopoietic stem cell transplantation (HSCT). Despite corticosteroids and ruxolitinib as first- and second-line therapies, up to 50% of patients develop refractory disease, with limited evidence guiding third-line interventions. Anti-thymocyte globulin (ATG), historically used in conditioning regimens, has shown variable efficacy in steroid-refractory aGVHD, but its role in patients previously exposed to ATG prophylaxis remains underexplored. Here, we report the case of a 19-year-old male with severe steroid- and ruxolitinib-refractory GI aGVHD, successfully treated with low-dose antithymocyte globulin (ATG) after failing multiple therapies (mycophenolate mofetil, anti-CD25 monoclonal antibody, mesenchymal stem cells, and methotrexate). This case underscores that, despite the prior use of ATG in the conditioning regimen and the multitude of available treatment options for refractory aGVHD, ATG can still be considered as a viable salvage therapy in situations where certain newer agents are not accessible.

## 1 Introduction

Acute graft-versus-host disease (aGVHD) remains the major cause of morbidity and mortality following allogeneic hematopoietic cell transplantation (HSCT), despite advances in prophylactic approaches (1). Acute graft-versus-host disease (aGVHD) predominantly manifests in the skin, gastrointestinal tract, and liver. High-dose corticosteroids are the standard first-line treatment for aGVHD (2). However, in ~35–50% of patients fail to respond (3, 4). Steroid-refractory aGVHD is associated with high mortality rates, as most patients succumbing to organ failure or infection within a few months (5, 6). The weighted average 6-month survival estimate derived from 25 studies was 49% (4). The gastrointestinal (GI) tract, serving as a critical target organ, plays a pivotal role in determining therapeutic outcomes, with patients manifesting GI involvement showing poor response to corticosteroids and higher mortality rates (7, 8). Until recently, there were no standard second-line treatments for steroid-resistant or steroid-dependent acute GVHD. The JAK2 inhibitor ruxolitinib, used as the currently recommended second-line treatment for steroid-refractory or steroid-dependent acute GVHD has shown notable efficacy, yet some patients develop resistance (9). A variety of therapeutic agents, including anti-tumor necrosis factor-alpha antibodies, anti-CD25 monoclonal antibodies, anti-IL-2R antibodies, mycophenolate mofetil (MMF), mesenchymal stem cells (MSCs), and anti-thymocyte globulin (ATG), are now administered as third-line options (5, 10, 11). None of these agents have demonstrated superiority over others in clinical practice. It is recommended to switch to an alternative drug if the initial therapy proves ineffective. The potential efficacy and clinical utility of these agents warrant further exploration.

ATG is a polyclonal antilymphocyte globulin that exerts immunomodulatory effects through multiple mechanisms, including T-cell depletion, induction of apoptosis in B-cell lineages, and the induction of regulatory T cells (Tregs) and natural killer T cells (12–14). During the early era of GVHD management, prior to the routine incorporation of ATG into prophylactic conditioning regimens, multiple clinical investigations explored its efficacy as a second-line treatment, particularly for steroid-refractory GVHD (14–16). However, with the increasing use of ATG in conditioning protocols for GVHD prevention, its application and exploration as a therapeutic option have progressively declined. Herein, we present a case of severe steroid- and ruxolitinib-refractory GI aGVHD that was successfully managed with ATG as salvage therapy.

## 2 Case presentation

**Figure 1:**
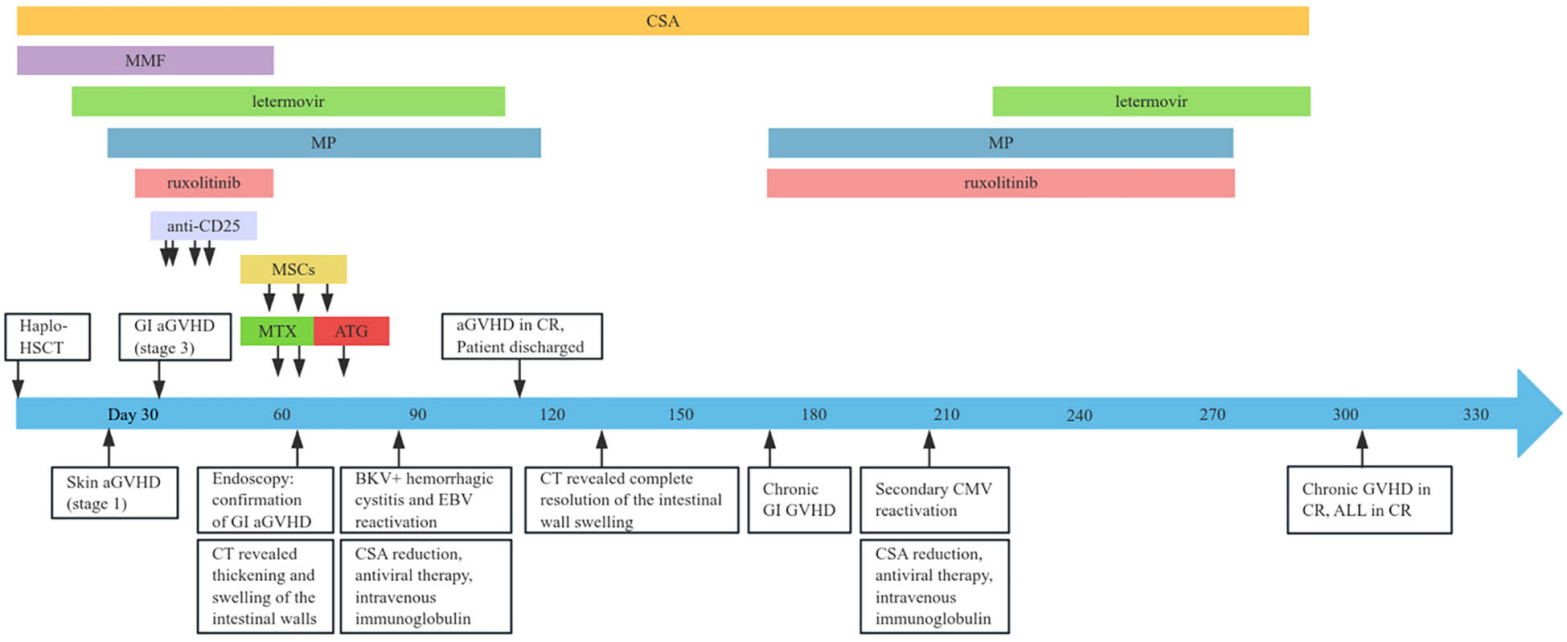
Clinical timeline outlining the disease course and therapeutic interventions. haplo-HSCT, haploidentical hematopoietic stem cell transplantation; aGVHD, acute graft-versus-host disease; GI, gastrointestinal; ATG, anti-thymocyte globulin; CMV, cytomegalovirus; CSA, cyclosporine; CR, complete remission; EBV, Epstein-Barr virus; MMF, mycophenolate mofetil; MP, methylprednisolone; MSCs, mesenchymal stem cells; MTX, methotrexate; ALL, acute lymphoblastic leukemia.

**Figure 2:**
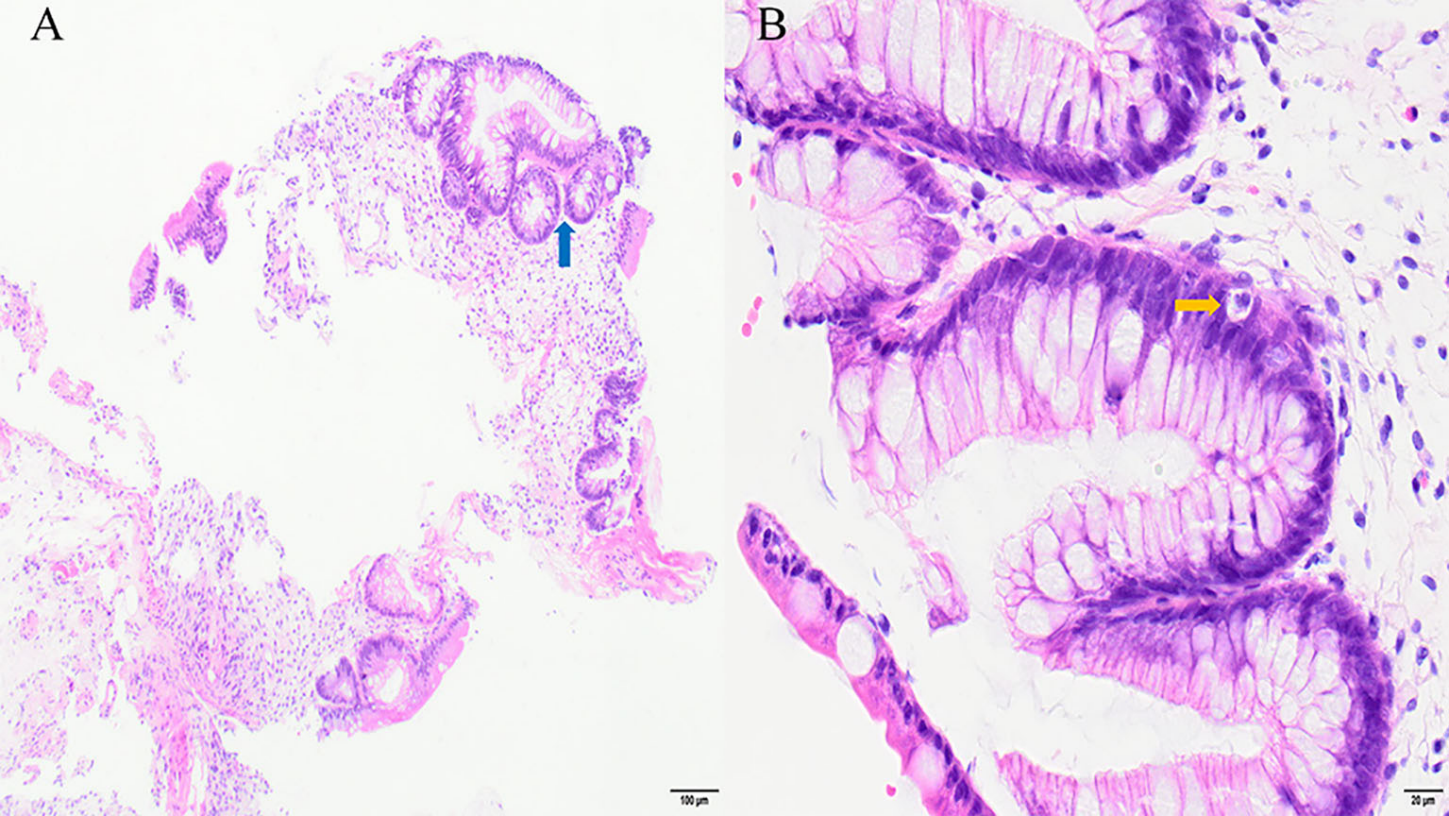
Ileocecal biopsy sample in stage 3 gastrointestinal acute GVHD: apoptotic epithelial cells (yellow arrow) and crypt loss (blue arrow). Hematoxylin and eosin stain: **(A)** ×100; **(B)** ×400.

A 19-year-old male with no significant comorbidities or relevant past medical history was diagnosed with high-risk Philadelphia chromosome-negative (Ph-negative) B-cell acute lymphoblastic leukemia (ALL). Following induction and consolidation chemotherapy, the patient underwent a haploidentical hematopoietic stem cell transplantation (haplo-HSCT) utilizing a myeloablative conditioning regimen on March 5, 2024. The donor was his brother, with a human leukocyte antigen (HLA) 6/10 allele match. Pre-transplantation screening revealed that the recipient had no donor-specific anti-HLA antibodies (DSAs); as a result, desensitization strategies were not deemed necessary. The conditioning regimen included busulfan (Bu) 3.2 mg/kg per day from days −7 to −4, cyclophosphamide (Cy) 60 mg/kg per day from days −3 to −2. Additionally, ATG was added to the regimen at a total dose of 10 mg/kg from days −5 to −2. aGVHD prophylaxis consisted of cyclosporine A (CsA), low-dose methotrexate (MTX), and MMF. The infused cell dose was 8.41×10^8^/kg nucleated cells and 6.20×10^6^/kg CD34+ cells. Neutrophil and platelet engraftment were achieved on day 13 and day 12, respectively. The patient received letermovir as cytomegalovirus (CMV) prophylaxis starting on day 12 of the treatment course. On day 21, he developed acute skin GVHD (stage 1), which was initially treated with methylprednisolone (MP) at a dose of 1mg/kg/day (**Figure 1**). Despite an increase in the MP dose to 1.5 mg/kg/day, the patient’s symptoms persisted. On day 26, oral ruxolitinib was introduced at a dose of 10 mg twice daily, following which the rash gradually resolved. On day 32, the patient developed abdominal pain and watery diarrhea, with a total volume of 1100 mL over seven episodes. Diagnosis of overall grade III aGVHD was made. There was no fever or abnormal function liver tests suggestive of aGVHD. Stool cultures were negative for bacterial, viral, or parasitic infections. Symptomatic treatment was ineffective, and the diarrhea volume persisted at 1000–1500 mL per day, accompanied by hematochezia. A diagnosis of steroid- and ruxolitinib-refractory GI GVHD (stage 3) was considered. Treatment with CsA, MMF, and ruxolitinib for GVHD was continued, and 4 doses of recombinant humanized anti-CD25 monoclonal antibody (1 mg/kg) were added, with the initial dose given on day 33 and subsequent doses on day 35, 40, 43, respectively. During this period, the symptoms of abdominal pain, diarrhea, and hematochezia transiently improved but subsequently worsened again. MMF and ruxolitinib were stopped on day 57 due to their lack of clinical efficacy and to mitigate the degree of immunosuppression. On day 60, MTX at a dose of 10 mg was introduced into the therapeutic regimen. This intervention resulted in transient amelioration of abdominal pain and hematochezia. However, the daily diarrhea volume remained high, ranging between 1500 and 2000 mL, and intermittent hematochezia persisted. On day 63, an abdominal computed tomography (CT) scan revealed thickening and swelling of the walls of the small intestine and colon. Concurrent endoscopic evaluation revealed diffuse mucosal erythema, erosions, and adherent dark red blood clots throughout the colon and terminal ileum. Histopathological examination of biopsies demonstrated apoptotic epithelial cells and crypt loss, confirming GI aGVHD (**Figures 2A, B**). On day 64, a second dose of MTX (10 mg) was administered. However, no clinical improvement was observed: diarrhea volume persisted at 1000–2000 mL per day, and hematochezia remained refractory to treatment. Due to an unsatisfactory response to previous treatment for aGVHD, the patient received three infusions of MSCs between days 57 and 71. Despite this, no significant clinical improvement was observed. On day 73, ATG at a dose of 3 mg/kg was added to the treatment regimen. The patient’s clinical course showed significant improvement, characterized by a progressive reduction in daily diarrhea volume to below 1000 mL, accompanied by significant improvement in abdominal pain and complete resolution of hematochezia. Over the subsequent month, bowel movements normalized. The patient developed BKV+ hemorrhagic cystitis on day 86, followed by Epstein-Barr virus (EBV) reactivation are on day 91, which were effectively controlled through CSA dose reduction, antiviral therapy, and intravenous immunoglobulin. The administration of letermovir was discontinued on Day 100 due to economic constraints. His clinical condition was deemed sufficiently stable for discharge on day 113. A follow-up CT scan on day 132 revealed complete resolution of the previously observed intestinal wall swelling. On day 170, the patient developed mild chronic GI GVHD manifesting as abdominal pain and low-volume diarrhea (<500 mL/day). The condition responded favorably to a combined immunosuppressive regimen comprising MP, CSA, and ruxolitinib. Notably, a secondary CMV reactivation occurred on day 206 during the course of GVHD treatment, which was effectively managed using a therapeutic strategy consistent with our previous protocol. Following confirmation of CMV negativity, letermovir was reintroduced as a prophylactic measure to prevent subsequent CMV infection and was continued until the discontinuation of immunosuppressive therapy. Throughout the entire clinical course, comprehensive surveillance revealed no additional infectious complications. The patient’s most recent bone marrow evaluation at 11-month post-transplant was negative for disease recurrence and full donor engraftment by mixed-chimerism analysis. His aGVHD remains quiescent, with no manifestations of chronic GVHD observed.

### 2.1 Cytokines status and lymphocyte immune signatures measurement

**Figure 3:**
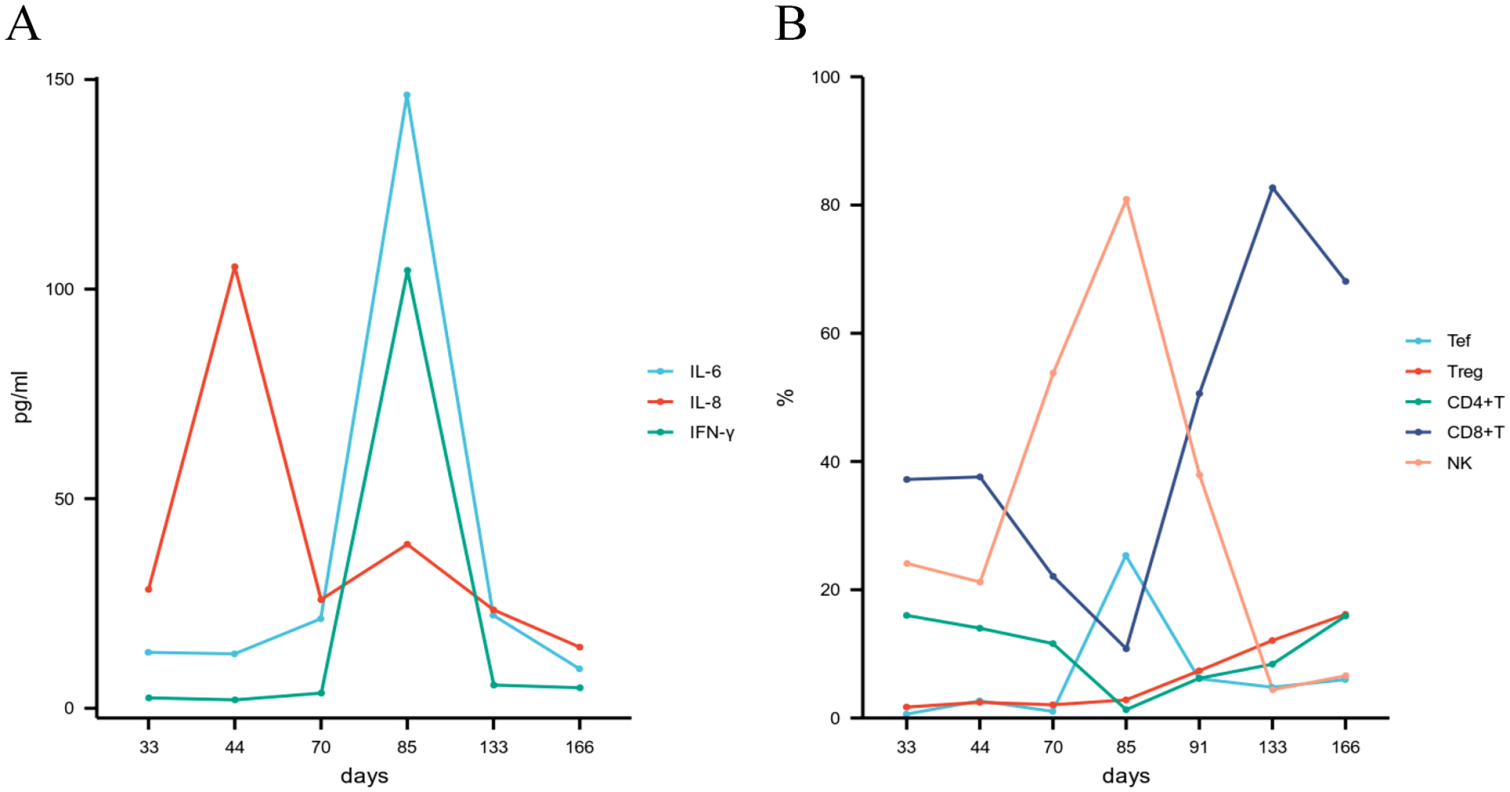
**(A)** Cytokine profiles; **(B)** Lymphocyte subset dynamics following ATG therapy. ATG, anti-thymocyte globulin; IL-6, interleukin-6; IL-8, interleukin-8; IFN-γ, interferon-gamma; NK, natural killer cell; Tef, effector T cell; Treg, regulatory T cell.

Using flow cytometry, we analyzed the dynamic changes in peripheral blood lymphocyte subsets pre- and post- ATG therapy, alongside the quantification of serum levels of interleukin-6 (IL-6), interleukin-8 (IL-8), and interferon-gamma (IFN-γ). We observed a rapid depletion of T cells, a sustained expansion of regulatory T (Treg) cells, and a transient increase followed by a subsequent decline in natural killer (NK) cells and effector T (Tef) cells following ATG administration. Furthermore, a significant increase in IL-6, IL-8, and IFN-γ levels was observed post-ATG treatment (**Figures 3A, B**). However, the potential influence of MP, MTX, and MSCs cannot be ruled out.

## 3 Discussion and conclusions

Steroid-refractory aGVHD is a critical post-transplant complication. Though ruxolitinib is the standard treatment, nearly half of patients still perish due to GVHD or related complications during GVHD treatment. In this case, we presented a patient who underwent haplo-HSCT following a myeloablative conditioning regimen incorporating ATG, and subsequently developed steroid- and ruxolitinib-refractory GI aGVHD. Despite treatment with MMF, anti-CD25 monoclonal antibody, MSCs, and MTX, the clinical outcome remained suboptimal. Notably, adjunctive administration of low-dose ATG resulted in significant alleviation of abdominal pain, diarrhea, and hematochezia. This finding highlights the potential utility of ATG as a salvage therapy, even in cases where ATG was previously incorporated into the conditioning regimen and where multimodal treatment strategies have proven ineffective.

ATG exerts its immunosuppressive effects by depleting lymphocytes in the peripheral circulation. Initial retrospective analyses have shown favorable response rates among patients treated with ATG or ATG + prednisone, contributing to the widespread adoption of ATG as a therapeutic option for steroid-refractory aGVHD (15, 16). However, some studies have suggested that it may not be an effective option for steroid-refractory aGVHD. For example, a study assessing long-term survival of patients with grade III/IV aGVHD found that among 34 steroid - refractory patients treated with ATG, 41% (14/34) had a response at 4 weeks, which declined to 21% (7/34) at 12 weeks. Skin aGVHD demonstrated the best response (80% at 4 weeks). 4 of them were alive at the time of the analysis (17). With the improvements of contemporary prophylaxis and treatment programs, recent studies have reported better outcomes with ATG. A small single-center study (n=11) demonstrated the efficacy of response-guided ATG therapy for steroid-refractory aGVHD. The overall response at day 28 was 55%. The 1 - year overall survival and transplant - related mortality were 55% and 45% respectively. The treatment did not increase the risk of opportunistic infections, and no cases of post-transplant lymphoproliferative disorder were observed (14). A phase 3, randomized trial comparing inolimomab (an anti–IL-2R antibody) with ATG found 40% (20/51) patients survived in the ATG arm (18). It is noteworthy that in most prior studies evaluating ATG for steroid-refractory aGVHD, the majority of patients had not received ATG as part of their conditioning regimens.

The core pathology of GI aGVHD lies in the persistent attack of donor T cells on host epithelial cells and the excessive release of pro-inflammatory cytokines (11). In this case, the depletion of peripheral T cells, the sustained expansion of Tregs, and the increase in pro-inflammatory cytokines (IL-6, IL-8, and IFN-γ) following ATG treatment were observed, which was consistent with previous research (13, 19–21). Despite the fact that elevated pro-inflammatory cytokines are typically associated with the development and progression of aGVHD (22). These factors increased following ATG treatment in this patient, while the symptoms of aGVHD significantly improved. This contradictory phenomenon may reflect the complex interplay between ATG’s immunomodulatory effects and the pathophysiology of aGVHD. During this period, the patient did not show any obvious infections; although the potential influences of MP, MTX, and MSCs cannot be ruled out, the temporal association between ATG administration and the fluctuations in cytokine levels suggests a core role for ATG. This phenomenon indicates that relying solely on cytokine levels to assess disease activity has its limitations, and a dynamic and comprehensive analysis of the immune status is necessary.

This study has several limitations. First, the patient received MTX and MSCs prior to ATG therapy, and the immunosuppressive effects of these treatments may have cumulatively influenced clinical outcomes, making it challenging to fully delineate the independent contribution of ATG. For example, MTX may enhance ATG’s lymphodepleting effects by suppressing T-cell proliferation, while the immunomodulatory properties of MSCs could further alter the intestinal microenvironment. Second, although symptomatic improvement was temporally associated with ATG administration and concurrent interventions showed no significant contemporaneous effects, the short observation period (11 months) and subsequent chronic GVHD and viral reactivation events highlight the need for longer-term follow-up to evaluate the durability of benefits from ATG. Future prospective studies are required to validate ATG’s efficacy in patients not receiving prior similar interventions and to explore dose optimization strategies.

In conclusion, this case highlights the effectiveness of ATG as a salvage therapy for steroid- and ruxolitinib-refractory GI aGVHD. The administration of low-dose ATG resulted in significant clinical improvement, including reduced diarrhea and resolution of hematochezia, despite prior treatment failures. This underscores the need to explore alternative therapies for refractory aGVHD cases. The observed immune dynamics, including elevated pro-inflammatory cytokines alongside symptom relief, indicate a complex interplay that necessitates a comprehensive management approach. Overall, this case enriches the discussion on innovative treatment strategies for challenging aGVHD cases.

## Data Availability

The raw data supporting the conclusions of this article will be made available by the authors, without undue reservation.
